# Histological and Molecular Evaluation of Patient-Derived Colorectal Cancer Explants

**DOI:** 10.1371/journal.pone.0038422

**Published:** 2012-06-04

**Authors:** Joshua M. Uronis, Takuya Osada, Shannon McCall, Xiao Yi Yang, Christopher Mantyh, Michael A. Morse, H. Kim Lyerly, Bryan M. Clary, David S. Hsu

**Affiliations:** 1 Institute for Genome Sciences and Policy, Duke University, Durham, North Carolina, United States of America; 2 Department of Surgery, Duke University, Durham, North Carolina, United States of America; 3 Department of Pathology, Duke University, Durham, North Carolina, United States of America; 4 Division of Medical Oncology, Duke University, Durham, North Carolina, United States of America; Huntsman Cancer Institute, University of Utah, United States of America

## Abstract

Mouse models have been developed to investigate colorectal cancer etiology and evaluate new anti-cancer therapies. While genetically engineered and carcinogen-induced mouse models have provided important information with regard to the mechanisms underlying the oncogenic process, tumor xenograft models remain the standard for the evaluation of new chemotherapy and targeted drug treatments for clinical use. However, it remains unclear to what extent explanted colorectal tumor tissues retain inherent pathological features over time. In this study, we have generated a panel of 27 patient-derived colorectal cancer explants (PDCCEs) by direct transplantation of human colorectal cancer tissues into NOD-SCID mice. Using this panel, we performed a comparison of histology, gene expression and mutation status between PDCCEs and the original human tissues from which they were derived. Our findings demonstrate that PDCCEs maintain key histological features, basic gene expression patterns and *KRAS*/*BRAF* mutation status through multiple passages. Altogether, these findings suggest that PDCCEs maintain similarity to the patient tumor from which they are derived and may have the potential to serve as a reliable preclinical model that can be incorporated into future strategies to optimize individual therapy for patients with colorectal cancer.

## Introduction

Colorectal cancer (CRC) is the third most common cancer and the second leading cause of cancer death in the United States. In 2010, approximately 142,000 people were diagnosed with CRC, and about 40% of these patients presented with advanced disease [1]. Treatment for advanced CRC with chemotherapy is typically intended for disease control and palliation of symptoms only, and as a result, unresectable CRC remains an incurable disease. In order to improve clinical outcomes and develop new therapeutic approaches, the development of a reliable preclinical model to study CRC biology and drug sensitivities is required.

Mouse models of CRC remain one of the most useful tools to decipher the biological mechanisms underlying the oncogenic process. To date, a variety of genetically-engineered, carcinogen-induced and xenograft mouse models have been established [2], [3] and it is generally agreed that no one model is sufficient to elucidate all aspects of CRC etiology.

Genetically engineered mouse (GEM) models have been invaluable in establishing the role of many different genetic mutations and signal transduction pathways contributing to the oncogenic process and allow investigation in the context of an active immune system [2], [3]. However, many of these GEM models, primarily those involving mutation of the *APC* tumor suppressor gene, develop tumors in the small intestine rather than the colon. This makes longitudinal disease progression studies difficult in addition to lacking the genetic complexity observed in human cancers [2], [3].

Another widely used mouse models of CRC relies on the use of carcinogens to induce colorectal tumor development. Perhaps the most widely used carcinogen-based model is the Azoxymethane (AOM) model. Here, colorectal tumor development is initiated by AOM, a potent, colon-specific carcinogen through the formation of DNA adducts [4]. Colorectal tumors derived using this model recapitulate key human pathological features observed in humans and allow investigation of the early stages of CRC. However tumor initiation and development is a time consuming process, often taking up to 6 months with tumor multiplicity and penetrance depending heavily on the mouse strain [2], [5], [6].

While GEM and carcinogen-based models have significantly enhanced our knowledge of the genetics and etiology of CRC, these models do not allow for accurate testing of cancer therapeutics to be used in the clinical setting [7]. The most widely utilized *in vivo* model for the testing of anti-cancer drug efficacy and combinations is the xenograft model. Historically, xenografts have been established through the subcutaneous injection of genetically-defined human-derived cell lines into immune-compromised nude mice [8]. However, to date, the majority of these cell line-based xenograft models have failed to generate drug sensitivity data that translates into clinically relevant information [7]. In addition, recent reports suggest that tumor-stroma interactions not present in cell line-based xenografts may represent an integral component in oncogenic potential and tumor drug response [9], [10]. Therefore, more recently, whole-tissue explants derived from human cancers including breast [11], lung [12], prostate [13] and colorectal cancer [14]–[16] have been established in an attempt to generate more clinically accurate and reliable xenograft models. However, these studies examined mainly early passage explants (<5 generations) from predominantly primary tumors and therefore there remains the need to further characterize these models and evaluate how well they retain important characteristics of the original human tumor especially in metastatic disease.

**Table 1:** Sites from which PDCCEs were derived.

Sample ID	Anatomic Location
CRC020	Liver
CRC025	Liver
CRC028	Colon
CRC030	Colon
CRC034	Liver
CRC039	Liver
CRC040	Liver
CRC043	Liver
CRC054	Liver
CRC057	Lung
CRC066	Colon
CRC075	Liver
CRC083	Lung
CRC093	Omentum
CRC096	Colon
CRC102	Liver
CRC103	Peritoneum
CRC105	Colon
CRC108	Liver
CRC119	Liver
CRC120	Liver
CRC133	Lung
CRC149	Liver
CRC159	Lung
CRC162	Liver
CRC167	Liver
CRC170	Liver

**Figure 1:**
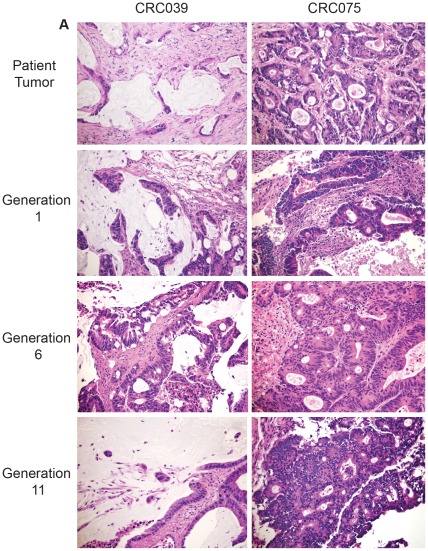
PDCCE tumor pathology is retained after 11 generations in mice. H&E stained sections of two independent well-differentiated adenocarcinomas (CRC039 and CRC075) show that tumor architecture remains similar after 11 passages in NOD/SCID mice. Images shown are at 20× magnification.

**Table 2:** Histological comparison of patient tumor and PDCCEs.

Sample ID	Source	Generation	%Tumor	%Necrosis	%Stroma	%Tumor Gland Formation	Histologic Type
CRC008	Patient		65	5	25	80	Adenocarcinoma, NOS
	PDCCE	3rd	70	10	20	70	Adenocarcinoma, NOS
CRC028	Patient		10	80	10	40	Adenocarcinoma w/signet ring
	PDCCE	1st	25	5	70	40	Adenocarcinoma w/signet ring
CRC034	Patient		65	0	35	80	Adenocarcinoma, NOS
	PDCCE	1st	85	5	10	75	Adenocarcinoma, NOS
CRC039	Patient		10	0	90	50	Adenocarcinoma w/mucinous
	PDCCE	1st	60	0	40	70	Adenocarcinoma w/mucinous
	PDCCE	6th	25	65	10	60	Adenocarcinoma w/mucinous
	PDCCE	10th	35	50	15	80	Adenocarcinoma w/mucinous
CRC057	Patient		25	45	20	80	Adenocarcinoma, NOS
	PDCCE	1st	40	60	0	80	Adenocarcinoma, NOS
	PDCCE	5th	50	35	15	80	Adenocarcinoma, NOS
CRC066	Patient		25	45	30	80	Adenocarcinoma, NOS
	PDCCE	1st	20	5	75	50	Adenocarcinoma, NOS
CRC075	Patient		35	30	25	90	Adenocarcinoma, NOS
	PDCCE	1st	50	15	35	95	Adenocarcinoma, NOS
	PDCCE	5th	85	5	10	75	Adenocarcinoma, NOS
	PDCCE	11th	65	10	25	70	Adenocarcinoma, NOS
CRC093	Patient		15	30	55	60	Adenocarcinoma, NOS
	PDCCE	1st	65	30	5	80	Adenocarcinoma, NOS
	PDCCE	5th	80	10	10	85	Adenocarcinoma, NOS
CRC096	Patient		20	65	15	65	Adenocarcinoma, NOS
	PDCCE	14th	65	30	5	90	Adenocarcinoma, NOS
CRC102	Patient		10	70	20	60	Adenocarcinoma, NOS
	PDCCE	1st	80	3	17	80	Adenocarcinoma, NOS
CRC103	Patient		5	40	55	100	Adenocarcinoma, NOS
	PDCCE	1st	10	90	0	95	Adenocarcinoma, NOS
	PDCCE	6th	10	90	0	95	Adenocarcinoma, NOS
CRC120	Patient		35	0	65	50	Adenocarcinoma w/signet ring & mucinous
	PDCCE	1st	85	0	15	70	Adenocarcinoma w/signet ring
CRC133	Patient		65	10	15	90	Adenocarcinoma, NOS
	PDCCE	7th	10	85	5	80	Adenocarcinoma, NOS
CRC149	Patient		25	60	15	80	Adenocarcinoma, NOS
	PDCCE	1st	40	55	5	70	Adenocarcinoma, NOS
CRC170	Patient		10	40	50	90	Adenocarcinoma, NOS
	PDCCE	2nd	80	5	15	80	Adenocarcinoma, NOS

**Figure 2:**
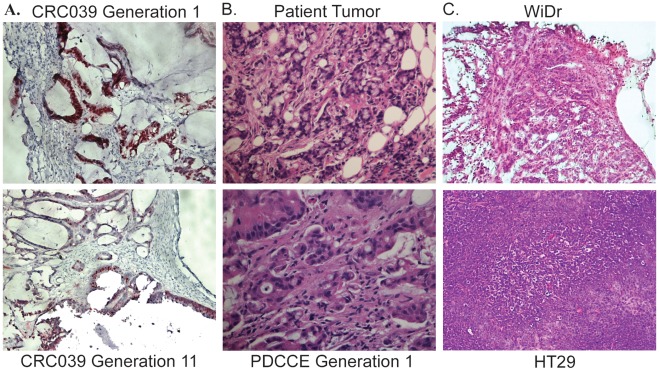
PDCCEs retain nuclear CDX2 expression and signet ring morphology observed in original patient tumors. A. Representative PDCCE (CRC039) retains nuclear CDX2 expression after 11 generations in mice. Images shown are at 20× magnification. B. Early passage PDCCEs retain signet ring morphology observed in original patient colorectal tumor. Images shown are at 40× magnification. C. Xenografts generated from WiDr and HT29 CRC cell lines lack histological features consistent with patient-derived explants including the presence of stroma and the formation of glands. Images shown are at 20× magnification.

In this study, we have performed a more comprehensive molecular and histological analysis of a panel of 27 matched patient-derived colorectal cancer explants (PDCCEs) from both primary and metastatic sites as an extension of our previous work [17] in which we compared the gene expression profile of 14 matched PDCCEs and their corresponding human tumors. We now demonstrate that PDCCEs retain global gene expression patterns, oncogene mutation status and histological parameters present in the original human cancers. Altogether these findings suggest that PDCCEs have the potential to serve as a reliable preclinical model that can be used to develop and characterize new therapeutic targets for patients with CRC.

## Materials and Methods

### Tumor Samples/Ethics Statement

A total of 27 human samples were obtained for genomic and histological analysis. All patients provided written consent to have tissue stored and used for research. Samples used for analysis in the laboratory were de-identified and not linked with any personal health information (PHI). All parts of this study were approved by the Duke Institutional Review Board. All animal studies were performed under a Duke University Institutional Animal Care and Use Committee (IACUC) approved protocol.

**Figure 3:**
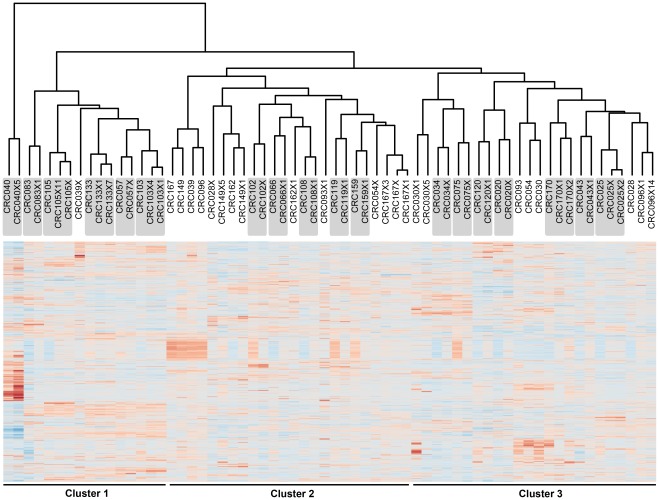
Patient tumor tissues and matched PDCCEs exhibit similar gene expression patterns. Unsupervised cluster analysis of 27 patient tumor-PDCCE matched pairs show that 22 pairs (81%) fell within the same cluster and 18 matched PDCCE (66%) clustered directly in pairs with the original patient tumor (gray boxes). Sample names containing an X denote PDCCE (xenograft) samples. The number immediately following the X indicates the generation/passage number of that particular sample.

### Generation of Patient-Derived Colorectal Cancer Explants (PDCCEs)

Colorectal tumors (both primary and metastatic) at time of surgery were collected under a Duke IRB approved protocol (Pro00002435). The tissues were washed with phosphate buffered saline (PBS) and then minced into pieces approximately ∼2 mm in size and injected into the flanks of 4-week-old NOD.CB17-PrkdcSCID-J mice obtained from Jackson Laboratories under a Duke IACUC approved protocol. Mice were observed and tumors measured with vernier calipers until the volume of the tumor ((V = L×2W×0.52 (L  =  longest diameter, W  =  shortest diameter)) reached ∼1,000 mm^3^. Tumors were then harvested, minced and re-implanted as described above until stable PDCCEs were established. At each generation, tumors were harvested and either fixed in 10% neutral buffered formalin (NBF), snap frozen in liquid nitrogen or frozen in optimal cutting temperature (OCT) medium on dry ice for further analysis.

**Table 3:** Patient-derived colorectal cancer explants’ *KRAS* and *BRAF* mutation status.

Sample ID	*KRAS* Status	*BRAF* status
CRC020	WT (patient); C12 GGT>GCT(mouse)	WT
CRC025	WT	WT
CRC028	WT	WT
CRC030	WT	WT
CRC034	WT	WT
CRC039	C12 GGT>GCT	WT
CRC040	WT	WT
CRC043	C12 GGT> AGT	WT
CRC054	C12 GGT>GAT	WT
CRC057	C12 GGT>GAT	WT
CRC066	WT	WT
CRC075	WT	WT
CRC083	C12 GGT>TGT	WT
CRC093	WT	WT
CRC096	C12 GGT>GCT	WT
CRC102	WT	WT
CRC103	C12 GGT>GTT	WT
CRC105	C13 GGC>GAC	WT
CRC108	WT	WT
CRC119	C12 GGT>GTT	WT
CRC120	WT	WT
CRC133	WT	WT
CRC149	WT	WT
CRC159	C12 GGT>AGT	WT
CRC162	WT	WT
CRC167	C12 GGT>GCT	WT
CRC170	C13 GGC>GAC	WT

### Histological Preparation and Examination

Paraffin-embedded PDCCE tissues were sectioned in 6 µm intervals and stained with hemotoxylin and eosin (H&E). Each sample was evaluated by a trained pathologist for the following histological criteria: histologic type, CDX-2 positivity, and relative percentage of tumor, necrosis, stroma, tumor gland formation and CDX-2 positive nuclei. All tissues were examined using >10 high-powered fields per section. Tumor nuclei were evaluated for CDX-2 staining using a standard quantitative scale of 0, 1+, 2+ and 3+. Staining of tumor nuclei at 2+ and 3+ was considered positive and all cases considered positive exhibited at least 20% of tumor nuclei with staining.

### Oncogene Mutation Analysis

Genomic DNA was isolated from snap frozen PDCCE tissues using a Qiagen genomic DNA isolation kit. Samples were diluted to 10 ng/µl and PCR was performed using the following primers for *KRAS*: forward 5′ GTGTGACATGTTCTAATATAGTCA 3′; reverse 5′ GAATGGTCCTGCACCAGTAA 3′ and *BRAF*: forward 5′ TCATAATGCTTGCTCTGATAGGA 3′; reverse 5′ GGCCAAAAATTTAATCAGTGGA 3′. Amplicons were sequenced by conventional methods using the forward primers.

### Microarray Analysis

RNA was isolated from snap-frozen PDCCE tissues using a Qiagen RNA/DNA Allprep kit, converted to cDNA and labeled by one cycle IVT. IVT labeled cDNAs were prepared according to the manufacturer’s instructions, and targets hybridized to the Human U133A 2.0 GeneChip and read on an Affymetrix array scanner. Raw data was converted to. CEL files and RMA normalized. CEL files (GSE35144) are available at the Gene Expression Omnibus (GEO) data repository (http://www.ncbi.nlm.nih.gov/geo/). To check for sample outliers and batch effects, 3D principal components analysis of the global gene expression was performed. Batch effects were normalized using the ComBat algorithm (http://jlab.byu.edu/ComBat/) [18]. Unsupervised hierarchical clustering of the human tumors and matching PDCCEs was performed on the 20% of genes with the greatest coefficient of variation. Agglomerative clusters were generated using the pearson correlation coefficient and complete linkage using the R program (The R Foundation for Statistical Computing).

### Software Used for Analysis

The R statistical software package is available at www.r-project.org. The Bioconductor R package is available at www.bioconductor.org. ComBat is available as an R script at http://jlab.byu.edu/ComBat/. Graphpad Prism is a product of Graphpad Software (La Jolla, CA, USA) and is available at www.graphpad.com/prism/prism.htm.

## Results

### Histological Evaluation of PDCCEs

A panel of 27 patient-derived colorectal cancer explants (PDCCEs) by direct transplantation of human colorectal cancer (CRC) tissues into NOD-SCID mice was created in this study. Table 1 shows the origin of the patient tumor and a total of 5 primary PDCCEs and 22 metastatic PDCCEs were generated. To assess the extent to which *in vivo* models of patient-derived colorectal cancer explants (PDCEEs) accurately recapitulate and can therefore serve as a model of the human condition, we investigated whether PDCCEs retain key biological features inherent to individual human colorectal cancers (CRC) over time. First, to evaluate the extent to which histological parameters are retained after xeno-transplantation, two independent PDCCEs were passaged through >10 generations and evaluated histologically. Both PDCCEs examined exhibited pathological features remarkably consistent with the original patient tumor through 11 generations (Figure 1). Next, a comprehensive histological evaluation performed on a sub-panel of 15 matched PDCCEs and original banked tissues revealed that 15/15 PDCCEs retained pathological features similar to those observed in the matched human tumor and were characterized as histologically identical to their matched original banked sample (Table 2). Even after 11 generations, PDCCEs retained the ability to form glands and contained CDX-2 positive nuclei comparable to the first generation PDCCEs (Figure 2). These data demonstrate that the histological features present in colorectal cancer, including the formation of glands and presence of stromal components are retained even in late passage explants, suggesting that unlike CRC cell line-derived xenografts, the PDCCE model provides us with a research tool that recapitulates the human condition generally not observed in other models.

### PDCCEs Retain Basic Global Gene Expression Profiles Inherent to Human Colorectal Cancers

Next, to further evaluate the extent to which PDCCEs represent their primary human counterparts, we analyzed 27 matched patient tumors and PDCCEs by microarray analysis. Patient tumor and PDCCE gene expression data was first normalized using ComBat to minimize batch effects. Unsupervised hierarchical clustering analysis was then performed on the normalized data set and revealed three distinct clusters (Figure 3). Of the 27 matched patient tumor and PDCCEs, 22 pairs (81%) fell within the same cluster based on the dendrogram and 18 PDCCEs (66%) clustered directly with the original tumor sample. Altogether, these data suggest that basic global gene-expression patterns are preserved between PDCCEs and their original human counterparts.

### Oncogene Mutation Status is Retained in PDCCEs

For patients with advanced colorectal cancer, the testing of mutation status of oncogenes such as *KRAS* is required for guiding therapy. Specifically, patients with *KRAS* mutations show no benefit from treatment with EGFR inhibitors such as cetuximab or panitumumab, while patients whose tumors are *KRAS* WT derive benefit from anti-EGFR based therapies [19], [20]. To determine whether these clinically-significant genomic parameters are maintained in PDCCEs, 27 matched PDCCEs and original patient samples were analyzed for *KRAS* and *BRAF* mutation status. Of the 27 matched pairs evaluated, 13 presented with activating *KRAS* mutations (codon 12 = 11; Codon 13 = 2) (Table 3). Of these 27 matched pairs, 26/27 PDCCEs (96%) matched their original human counterpart suggesting that human colorectal cancer tissues maintained as mouse PDCCEs are genetically stable and retain oncogenic mutation status critical to CRC pathophysiology. All samples tested negative for *BRAF* mutations. Altogether, these data suggest that PDCCEs maintain the biologically complex histological, gene expression and mutation-based characteristics observed in human CRCs.

## Discussion

To date, a number of mouse xenograft models have been established to investigate CRC etiology and treatment. To a large extent, these models have been generated using late passage cell lines derived from human CRCs and while significant treatment-induced tumor responses have been observed in these models, they are rarely predictive of tumor response in human patients [7]. This is likely due in these models, at least in part, to the inherent lack of stroma in tumor-derived epithelial cell lines. Mounting evidence indicates that paracrine signaling and extracellular matrix components supplied by neighboring stromal cells play a significant role in the oncogenic potential of colorectal carcinoma and that modulation of these stromal interactions directly impact the efficacy of chemotherapy on tumor response [9], [10].

Recent attempts have been made to generate mouse xenograft models of CRC by direct transplantation of human colon tumors into immune-compromised mice [14]–[16], [21]. Poupon and coworkers reported that the passage of human colon cancer tissues through a xenograft stage significantly improves the success rate of cell line derivation from human CRC metastases [15]. More recently, Hohenadl and coworkers reported that histological characteristics and oncogene expression levels are retained in early passage CRC xenografts [21] while Fichtner et al., and Messersmith et al, used panels of 15- and 10 human CRC explants respectively to evaluate drug sensitivity [14], [16]. Additionally, these studies used patient-derived explants mainly from the primary site and as metastatic tumors tend to be more aggressive and are more likely to differentiate, it remains unclear if PDCCEs generated from metastatic sites would maintain similarity to the original tumor. While these studies have begun to underscore the value of explant models in CRC research, a more comprehensive histological and molecular analysis on a larger panel of human CRC explants are needed to justify their use as a preclinical model to perform accurate drug efficacy analysis and predictive biomarker identification.

In this study, we demonstrate that PDCCEs generated from human adenocarcinomas with varying histological features each retain the parameters of the tumor from which they were derived at the histological, global RNA expression and oncogene mutation levels. Despite the existence of differences in the percentage of tumor stroma present between the original human tissues and those xenografted into mice, our study focuses on the malignant epithelial cells. First, the histological architecture inherent to colorectal adenocarcinoma, primarily the ability to form dysplastic glands as well as the presence of CDX-2 positive nuclei is maintained in the PDCCEs throughout multiple passages (>10). Next, we compared the gene expression profiles between matched PDCCEs and its corresponding patient tumor and observed that 18/27 (66%) of the samples clustered directly together and 22/27 (81%) clustered within the major cluster as defined by the dendrogram.

We speculate that the 9 PDCCE samples that did not cluster directly with their corresponding original tumor may have been due to the inherent heterogeneous nature of CRC. It is plausible that the original CRC tumor samples corresponding to these 9 PDCCEs harbored small sub-populations bearing additional oncogenic events. This would in turn confer a growth advantage to these populations after being transplanted into the mouse, causing the PDCCE to have a different genetic composition than the original tissue from which it was derived. In support of this notion, it appears that most variation between the primary tumor and its PDCCE occur in early PDCCE passages and that less variation occurs through the process of passaging. For example, PDCCE CRC105 clustered with the original patient sample at PDCCE passages 1 and 11 while PDCCE CRC149 did not cluster with its original sample at either passage 1 or 5 suggesting that genetic changes occur predominantly in early passages and are maintained through later passages. It is also possible, that in these 9 samples, there may have been a greater stromal contamination resulting in a difference in their clustering pattern. These results suggest that there are indeed intrinsic differences between the matched patient tumor and PDCCE and extrapolations drawn from these models must be done so carefully. However, overall our findings suggest that the PDCCE model has the potential to be used in the investigation of new therapeutic agents that target both the malignant epithelial tumor architecture and/or stromal component and that PDCCEs can be maintained for 10 or more generations while retaining key histological parameters.

Finally, we evaluated the *KRAS* and *BRAF* mutation status of the PDCCEs and showed that the status of all but one of the oncogene mutations was retained. We observed one case (CRC020) in which a *KRAS* activating mutation was present in the PDCCE but was not detected in the original patient samples despite the fact that these samples clustered together by unsupervised cluster analysis. It is most likely that a small, undetectable population of *KRAS* mutant cells was present in the patients tumor at the time of surgical resection and that the growth advantage conferred by *KRAS* activation allowed for subsequent expansion of *KRAS* mutant cells during early PDCCE passages.

Although any single mouse model will never fully recapitulate actual findings in patients, the use of preclinical models is necessary and practical for the development of therapeutic agents and biomarkers and a crucial first step in bringing these agents to the clinic. We do realize the limitations of our model and that any finding must undergo rigorous testing to gauge its accuracy, reliability, and reproducibility and must also be retrospectively validated in multiple patient samples. Nevertheless we feel that our preclinical mouse model has the potential to be used to identify and test novel combinations of therapeutic agents and to also develop both predictive and prognostic biomarkers, which can then be systematically brought forth into the clinical setting.
